# Gender Differences in Age-Related Changes in Cardiac Autonomic Nervous Function

**DOI:** 10.1155/2012/679345

**Published:** 2011-11-30

**Authors:** Shailaja Moodithaya, Sandhya T. Avadhany

**Affiliations:** ^1^Department of Physiology, K.S Hedge Medical Academy, Nitte University, Mangalore 575018, India; ^2^Department of Physiology, St. John's Medical College, Bangalore 560034, India

## Abstract

Ageing is associated with changes in cardiac autonomic control as measured by Heart Rate Variability (HRV). Not many studies have explored the influence of gender on age-related changes in cardiac autonomic regulation. This study evaluated the gender differences in age-associated changes in cardiac autonomic nervous activity by assessing HRV using frequency domain analysis of short-term stationary R-R intervals. HRV was studied in healthy males and females ranging in age from 6 to 55 years. Total power and absolute power in High-Frequency (HF) and Low-Frequency (LF) components as well as HF in normalized unit declined significantly with ageing. The HF/LF ratio was significantly higher in the adolescent and adult females compared to male of these age groups. This study suggests that gender differences exist in age-related changes in HRV. The finding that gender differences are limited to adolescent and adult age groups may indicate a role for female sex hormones in cardiac autonomic modulation.

## 1. Introduction

The autonomic nervous system plays a major role in the regulation of the cardiovascular system under both physiological and pathological conditions [1]. Analysis of the beat to beat variability of cardiac R-R intervals has been used to quantify alteration in cardiac autonomic regulation and also to predict adverse clinical events [2]. Periodic fluctuations of heart rate are indicative of the relative contributions of sympathetic and parasympathetic components of autonomic nervous system to heart. Spectral analysis of HRV studies the frequency-specific oscillations of heart rate fluctuation and decomposes series of sequential R-R intervals into a sum of sinusoidal functions of different amplitudes and frequencies. The power spectrum has shown at least two frequency bands: low frequency (LF) from 0.04 to 0.15 Hz and High frequency (HF) from 0.15 to 0.4 Hz. The HF fluctuation of R-R intervals mainly reflects the cardiovagal modulation and the inspiratory inhibition of vagal tone, whereas the LF bands influenced baroreceptor-mediated regulation of blood pressure and reflect predominantly sympathetic activity. The ratio of LF/HF has been used to reflect the sympathovagal balance [3, 4].

Normal human ageing is associated with changes in the autonomic control of several biological functions. Orthostatic maneuver such as going from supine to standing may trigger dizziness more frequently with ageing, reflecting the diminished cardiovascular sympathetic modulation. Similarly, the recovery of heart rate after exercise becomes blunted with age as a result of sluggish cardiac vagal response to adjust the cardiac activity. The two subsystems sympathetic and parasympathetic of autonomic nervous system mature with time, but degree of the changes due to ageing is different because of their divergent neural pathways. Therefore symaptho-vagal balance also fluctuates with ageing [5]. Autonomic nervous activity in relation to ageing was studied using conventional cardiac autonomic function tests. Age-related reductions in overall heart rate variation in response to deep breathing and Valsalva maneuver suggest that ageing is associated with impaired vagal control of heart rate [6]. Most of the currently available data on the effect of age on autonomic nervous function is derived from studies of HRV, reporting that increasing age is associated with a reduction in overall HRV [7, 8]. A study on confounding variables of cardiovascular autonomic function had revealed that age is one of the important confounders for cardiac autonomic function [9].

There are a few reports on gender-related differences in cardiac autonomic tone. In a population of normal subjects, using short-term and long-term recordings, there was found predominance of parasympathetic over sympathetic tone in women and vice versa in men. It was also demonstrated that the gender-related difference in parasympathetic regulation diminishes after the age of 50, while sympathetic dominance in men disappears significantly later [10]. Yukishita et al. reported that in general autonomic activities attenuate with age in both genders [11]. However, the influence of gender on age-related changes in cardiac autonomic activity is not well established. Therefore this study evaluated the gender differences in age-associated changes in cardiac autonomic nervous activity by assessing HRV.

## 2. Materials and Methods

A total of 267 healthy volunteers of which 126 males and 141 females between the age group of 6–55 years were recruited. The subjects were from wide range of social and economic classes. The participants were students and faculty of the Institute in Bangalore, South India. Study population was classified into eight groups based on age and gender. That is, men and women are in four age groups: children (6–11 yrs), adolescents (12–19 yrs), Young adults (20–40 yrs), and middle aged (41–55 yrs). Subjects belonging to all the study groups were well nourished based on their body mass Index. The female subjects included in children and middle-aged group were prepubertal and postmenopausal respectively. Menstruating female subjects participated were controlled for menstrual cycle and they were recruited during mid-follicular phase of their menstrual cycle. Subjects were screened after taking detailed medical history and measuring basal blood pressure. Subjects with hypertension and history of diabetes mellitus and any other chronic illness were excluded from the study. Subjects on oral contraceptive pill, hormonal replacement therapy, and drugs that alter the cardiovascular functions were also excluded from the study. Informed written consent was obtained from all participants and in case of children the parental consent was also obtained. The experiment protocol was approved by Ethics committee of the Institute.

All the experiments were conducted in the morning after overnight fasting. Subjects refrained from smoking, caffeinated beverages for at least 12 hours prior to the experiment, and also instructed to avoid strenuous physical activity from the previous evening.

### 2.1. Assessment of HRV

To quantify heart rate, the analog ECG signal was obtained using lead II to obtain a QRS complex of sufficient amplitude and stable base line. ECG signals were conveyed through an A/D converter (Biopac MP 30, Biopac system INC. Santa Barbara, CA) at a sampling frequency of 500 Hz to PC and were analyzed offline after visual checking of abnormal ECG. Heart rate variation during normal breathing for a period of 5 minutes was recorded, with subject supine, awake, and resting. The data gathered was subjected to frequency domain analysis of HRV.

Frequency domain analysis was performed using nonparametric method of Fast Fourier Transformation. Data was edited manually for artifacts and ectopic beats. HRV software used a peak detection algorithm to find the “R” wave, which was done at a resampling rate of “4 Hz”. A minimum of 256 data points was required to perform a spectral analysis. To attain 256 data points a duration of 5 minutes of ECG recording was required. The linear trend was removed from each data set to avoid its contribution to low-frequency power. The power frequency spectrum was subsequently quantified into standard frequency-domain measurements including total variance, HF (0.15–0.4 Hz), LF (0.04–0.15 Hz), and HF/LF ratio.

Following the ECG recording, and daily physical activity level was estimated using a validated physical activity questionnaire which assessed physical activity pattern over the preceding month [12]. A composite index of the level of activity was computed as the physical activity level, which is the 24-hour energy expenditure divided by the estimated basal metabolic rate. Physical activity level cutoff values for grades of physical activity have been defined previously: <1.4 sedentary; 1.55–1.6 moderately active; and >1.75 very active [13].

Statistical analysis was performed with an SPSS package (version 10.5). Normality of the distribution was assessed with the Kolmogorov-Smirnov goodness of fit test. Because of skewed distribution of absolute values of spectral powers, they were also analyzed after logarithmic transformation. Data is expressed as mean ± SEM with 95% confidence interval. All the variables were compared between age and gender groups using Two-Way ANOVA, with the test for age and gender effects as well as their interaction. Looking for the overlap of the confidence interval, the study assessed the differences between specific groups. Variables for which age-gender interaction exists and the effect of age amongst the specific groups were compared independently for males and females. For the remaining variables, comparison was done using combined values. In all instances null hypothesis was rejected at **P** < 0.05.

## 3. Results

Of the 267 healthy volunteers (126 males and 141 females) originally enrolled in the study, 15 were excluded due to reasons like presence of frequent premature ventricular beats, experience of syncope during ECG recording, and inability of the software to analyse the HRV.

Table 1 shows subject characteristics of the study population. Analysis using two-way ANOVA has shown that Resting Heart Rate (RHR) declined significantly with ageing (*P* < 0.01). Females had higher RHR than males in all the age groups except for the middle-aged subjects; however, difference in HR between males and females did not show any statistical significance. Systolic BP and Diastolic BP showed increasing trend with ageing (*P* < 0.01). SBP and DBP were significantly lower in females than males across all the age groups (*P* < 0.01). Physical activity level did not show any difference between the study groups.

Table 2 shows HRV spectral power in males and females of different age groups.

Total power and absolute power in HF and LF components HF in normalized unit as well as HF/LF declined significantly with ageing (*P* < 0.001). Normalized power in LF showed significant increase with ageing (*P* < 0.001). Absolute power in LF band (*P* = 0.034) and the HF/LF ratio (*P* < 0.001) were significantly higher in the adolescent and adult females compared to male of these age groups. There were no such gender differences in children and middle-aged subjects. HF/LF also exhibited a significant age and gender interaction (*P* = 0.035).

## 4. Discussion

The present study examined the effect of gender on age associated changes in cardiac autonomic activity. Autonomic regulation of heart rate was assessed by frequency domain analysis of short-term HRV at supine resting condition.

The findings of this study indicate that healthy ageing is associated with gradual reduction of overall fluctuation in autonomic input to the heart as well as vagal index of HRV which are reflected by significant decline in total power and HF in absolute power (Table 2). The higher relative power of LF and lower ratio of HF to LF from children age group to middle age suggest that ageing is associated with shifting cardiac autonomic tone towards sympathetic dominance. The decline in HRV indices was not significant from children age group to adolescent age groups for both females and males. An accelerated decline of HF was observed from adolescent to adult age (20–40 yrs) group and from adult to middle age (41–55 yrs).

**Figure 1:**
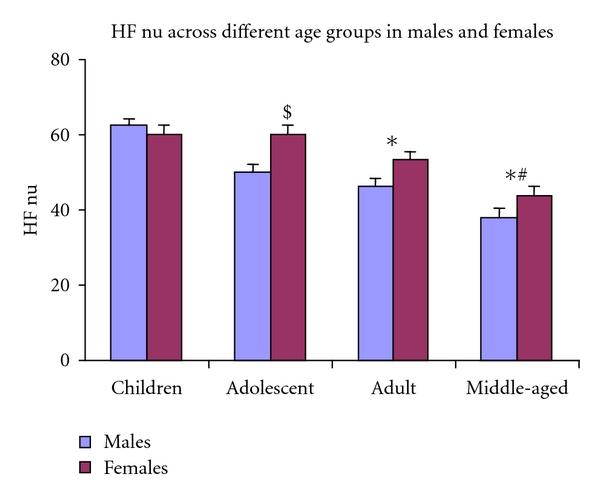
The figure shows that age-related decline of HF nu across four age groups in both males and females as well as gender-related difference in adolescent age group. *Significantly different from children group; ^#^Significantly different from adult group. ^$^Significantly different from males using two-way ANOVA.

**Figure 2:**
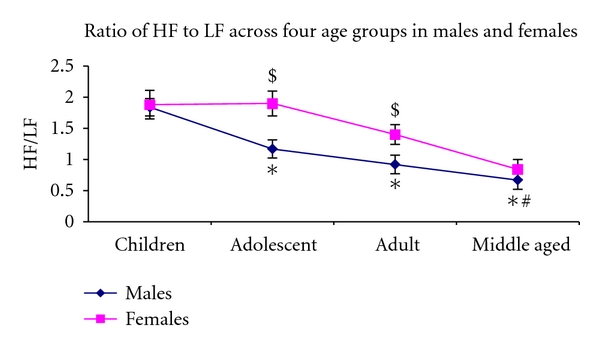
The figure shows ratio of HF to LF ratio across four age groups in males and females. It depicts the age-related decline in HF/LF ratio in males and females. Females have significantly higher HF/LF ratio as compared to males in the adolescent and adult groups. In the two extreme of ages the ratio is almost similar; *significantly different from children group; ^#^significantly different from adult group; ^$^Significantly different from males of similar age group using two-way ANOVA.

Reduction in HF nu and the ratio of HF/LF with ageing exhibited a different pattern in males and females (Figures 1 and 2). These indices were relatively stable from children group to adult age group, and prominent decline was observed from adult to middle-aged group in females. But among males, the reduction in these indices followed similar pattern like absolute power HF.

 The analysis of gender-related difference in HRV study indicates that HF in relative value (nu) and HF/LF ratio were significantly higher in women. However, HF component of HRV, denoting vagal modulation to heart, was not significantly different between men and women, when expressed in absolute value. Total power of HRV also did not differ between males and females; further LF in normalized and absolute values was significantly lower in females. The data also shows that the gender differences for these indices are age dependent, since differences exist for HF nu, only in adolescent group subjects, and for HF/LF ratio only in adolescent and adult age groups.

 The higher HF (nu) and HF/LF ratio observed in the female subjects of this study can be attributed to lower LF in absolute and relative values in females. It has been suggested that normalized powers were superior at detecting the effect of gender and these indices describe a balance, rather than a modulation of individual limbs of ANS [10]. All the HRV indices in this study did not show significant differences between males and females in children as well as middle-aged subjects, indicating that female sex hormones might influence autonomic balance, which is reflected by presence of gender difference in HF nu and HF/LF ratio in adolescent and adult age strata, in which subjects were of postpubertal and premenopausal females, respectively.

In this study for the first time the gender differences in cardiac autonomic nervous function in a population of wide age range were studied. Gonadal hormones play an important role in mediating gender differences observed in many physiological parameters. It is assumed that menopause constitutes a significant mile stone in terms of changes in cardiovascular physiology and risk for developing cardiovascular diseases. A gender difference in risk of developing cardiovascular disease tends to diminish in the postmenopausal age groups. Therefore, we chose the postmenopausal age as the upper cut-off for subject recruitment into this study. Further, as this study focused on healthy volunteers, recruitment of subjects above 55 years would have resulted in interference of many altered physiological conditions associated with ageing. It has been reported that autonomic function is modified in altered nutritional status [14]. All the subjects who participated in this study were well nourished, based on their BMI [15]. Since, the daily physical activity level is considered as one of the potential confounders in the measurement of autonomic nerve activity [16], the study groups were controlled for the physical activity levels in this study. Further, menstruating female subjects participated in this study were controlled for menstrual cycle, and they were studied during the mid-follicular of the menstrual cycle to observe the gender effect. The female subjects in children and middle-aged group were prepubertal and postmenopausal, respectively.

Measurements of HRV components, namely, HF in absolute power represent parasympathetic control and the LF represents both sympathetic and parasympathetic modulation to heart. Relative measurements (HF nu, LF nu) provide quantitative evaluation of graded changes in the state of parasympathetic and sympathetic modulation [3]. The HF/LF ratio provides an index of parasympathetic relative to sympathetic nervous system tone [17]. Microneurographic recordings of muscle sympathetic nerve activity (MSNA) offer a direct measurement of efferent postganglionic sympathetic nerve activity, which is considered as gold standard measurement of global sympathetic outflow to skeletal muscle, whereas HRV provides indirect indices of autonomic modulation. However, because of the invasive and complexity associated with the technique microneurography is not practical for studies involving large sample. Further, studies have shown that MSNA and cardiac sympathetic marker of HRV may change parallel in response to an autonomic challenge [18].

The gender-related differences in autonomic nervous system have been studied earlier. Most of the existing data on gender-related differences for power spectral variables belong to the subjects of age group above 40 years [19]. Umetani et al. defined the effect of gender, on normal range of HRV, over nine decades (9–100 yrs) in healthy subjects; however this study was based on time domain analysis. In this study authors reported that HRV for all time-domain measurements is lower in women especially below the age of 50 years and level of parasympathetic activity is lower in young women [20]. Kuo et al. demonstrated that the gender-related difference in parasympathetic regulation diminishes after age 50 yrs, based on the study of normal humans between 40 and 79 yrs of age. The same study also reported that women had a higher HF in the age strata of 40–49 yrs [10]. Results on HRV in the children group in this study are in agreement with the report of Galeev et al. in which they observed similar HRV for both males and females among children [21]. Higher HF nu and HF/LF ratio as well as lower absolute and normalized LF observed for females of adolescent and adult group in this study was comparable with the findings of Ramaekers et al., which showed that cardiac autonomic modulation is significantly lower in healthy women compared to men because of lower sympathetic activity [22]. The HF/LF ratio observed for middle-aged subjects for both males and females was similar in this study, which does not match with the findings of Liao et al. and Bigger et al. [7, 23]. The reason for this could be explained as a result of noncategorising the middle aged females, in their study into pre- and postmenopausal subjects.

The mechanism for gender differences in age-associated changes in cardiac autonomic function is obscure. Differences in the autonomic system may be due to differences in afferent receptor stimulation, in central reflex transmission, in the efferent nervous system, and in postsynaptic signaling. At each of these potential sites of difference, there may be effects due to different size or number of neurons, variations in receptors, differences in neurotransmitter content or metabolism, as well as functional differences in the various components of the reflex arc.

Ageing is associated with an increased dependency on sympathetic control of cardiac responses and reduced vagal responsiveness. The blunted vagal modulation of the heart may be related to altered neural vagal discharge to sino-atrial node or to a change in the ability of the cardiac pacemaker itself. Cardiac electrophysiological studies have demonstrated a progressive decline in sino-atrial conduction and sinus node recovery time with age. Studies have revealed an increase in empty Schwan cell bands or reduced number of fibers in the vagus nerve among old subjects [24]. Altered autonomic modulation with ageing also can be explained by dysfunction of baroreceptor mechanism. Increase in circulating levels of norepinephrine and thereby sympathetic over activity might account for reduced vagal efferent drive in advancing age [25].

A few studies have indicated that female sex hormones influence autonomic modulation and estrogen has a facilitating effect on cardiac vagal function [26]. The higher HF nu and HF/LF ratio observed in the female subjects can be attributed to lower sympathetic activity in females which is reflected by lower LF in absolute and relative values in females. This finding is in agreement with the findings of Ramaekers et al., where the authors postulated that lower sympathetic activity in females compared to men might provide an explanation for the protection against cardiovascular disease observed in females [22]. The current study results show a disappearance of gender differences in all HRV components among middle-aged subjects. Because all the women participated were postmenopausal in this age range, it is speculated that protective factor may be the female hormone estrogen. Potential mechanism possibly may include direct or indirect hormonal effects on electrical properties of sinus node or chronic developmental differences in sinus node.

**Strength of the Study**

For assessing gender differences in age-related changes in autonomic modulation, the current study included large sample size subjects from children to middle age, where stratified agewise classification is continuous. As altered nutrition, physical activity level, and phases of menstrual cycle are important confounding variables in measurement of heart rate variability, in the current study, the various study groups were controlled for the above-mentioned factors.

**Limitations of the Study**

Estimation of gonadal hormones and correlation of its levels with HRV indices would have provided better understanding of influence of hormones on gender differences in age-related changes cardiac autonomic activity. The current study has not estimated the gonadal hormonal levels of the participants. Though menopause is significant mile stone in cardiovascular research, recruitment of subjects above 55 years would better explain age-related changes in autonomic function.

**Implication of the Study**

Outcome of this study reveals that the assessment of sympathetic and parasympathetic nervous activity for risk stratification of autonomic related disorder should control for variables like age and gender. Considering physiological differences in evaluation of autonomic dysfunction would be useful for diagnosis and treatment of autonomic-related diseases.

**Future Research**

The outcomes of the study indicate that there is scope for further studies on investigation of autonomic nervous activity using sensitive techniques like MSNA and also evaluation of autonomic function in multiple organ systems to provide an index of age-related global autonomic nervous modulation.

**Conclusion**

This study concludes that gender differences exist in age-related changes in HRV. The finding that gender differences are limited to adolescent and adult age groups may indicate a role for female sex hormones in cardiac autonomic modulation. The exact impact of the neurohormonal axis on the age-related changes in cardiac autonomic nervous system remains to be elucidated.

## Figures and Tables

**Table 1:** Subject characteristics.

Parameter	Children		Adolescents		Young adults		Middle-aged	
	Males	Females	Males	Females	Males	Females	Males	Females
	(*n* = 30)	(*n* = 30)	(*n* = 29)	(*n* = 37)	(*n* = 30)	(*n* = 40)	(*n* = 29)	(*n* = 27)
Age (yrs)	9.4 ± 0.32	9.4 ± 0.3	17.0 ± 0.38	16.4 ± 0.38	29 ± 0.8	30.1 ± 0.86	48.3 ± 0.6	48.5 ± 0.64
Height (cm)	131 ± 1.98	130 ± 1.9	159 ± 5.1	154 ± 0.78	166 ± 1	156 ± 0.8	167 ± 3.8	155 ± 0.78
Weight (kg)*	27 ± 1.2	26.4 ± 1.9	55.5 ± 1.9	49.0 ± 1.1	59.4 ± 1.3	53.1 ± 0.95	58.9 ± 1.3	56.1 ± 0.9
SBP^∗#^	112 ± 1.6	108 ± 1.3	114 ± 2.3	109 ± 1.1	116 ± 1.1	111 ± 6.8	120 ± 1.66	118 ± 1.73
DBP^∗#^	74 ± 1.4	74 ± 0.76	76 ± 0.8	75 ± 0.7	78 ± 0.8	76 ± 0.7	84 ± 1.1	81 ± 1.2
RHR*	78 ± 1.8	82 ± 1.7	67 ± 1.96	69 ± 1.1	64 ± 1.7	67 ± 1.1	69 ± 1.96	66 ± 1.3
BMI*	15.5 ± 0.24	15.6 ± 0.22	20.5 ± 0.36	20.5 ± 0.37	21.6 ± 0.33	21.7 ± 0.33	22.1 ± 0.3	23.1 ± 0.3
PAL	N.A^@^	N.A^@^	1.64 ± 0.02	1.59 ± 0.02	1.6 ± 0.02	1.65 ± 0.02	1.65 ± 0.02	1.69 ± 0.02

(i) Data expressed as mean ± SE.

(ii) *significantly different across the age group.

(iii) ^#^significantly different between males and females.

(iv) SBP: Systolic blood pressure (mm of Hg).

(v) DBP: Diastolic blood pressure (mm of Hg).

(vi) RHR: Resting heart rate (Beats per minute).

(vii) BMI: Body Mass Index (kg/m^2^).

(viii) PAL: Physical activity level.

(ix) ^@^Not applicable since standard procedure was not available.

**Table 2:** Comparison of heart rate variability spectral power (absolute power) in males and females of different age groups (*n* = 252).

	Children	Adolescent	Young adults	Middle-aged
Total power (0–0.4 Hz) (ms^2^)				
Males	2694 ± 336	2072 ± 306	1171 ± 212	563 ± 121
	(2006–3381)	(1446–2697)	(736–1606)	(313–814)
Females	2433 ± 331	1612 ± 246	1148 ± 149	478 ± 82
	(1756–3110)	(1112–2112)	(846–1456)	(308–448)
Total	2564 ± 234	1822 ± 194	1158 ± 124^∗†^	520 ± 72^∗†‡^
	(2094–3033)	(1434–2209)	(909–1407)	(375–665)

Two-way ANOVA: *P* < 0.001 between age groups, *P* = 0.420 between genders, and *P* = 0.379 for interaction between age and gender.				

High-frequency power (0.15–0.4 Hz) (ms^2^)				
Males	1645 ± 209	1057 ± 165	533 ± 98	238 ± 56
	(1215–2074)	(720–1394)	(331–734)	(121–355)
Females	1561 ± 254	999 ± 155	628 ± 98	209 ± 56
	(1040–2082)	(683–1314)	(427–825)	(128–289)
Total	1603 ± 163	1025 ± 112	585 ± 69^∗†^	223 ± 34^∗†‡^
	(1250–1931)	(801–1275)	(446–724)	(155–291)

Two-way ANOVA: *P* < 0.001 between age groups, *P* = 0.995 between genders, and *P* = 0.592 for interaction between age and gender.				

Low-frequency power (0.04–0.15 Hz) (ms^2^)				
Males	1049 ± 148	1001 ± 187	638 ± 118	325 ± 66
	(745–1352)	(619–1384)	(396–879)	(188–461)
Females	872 ± 108	584 ± 111	522 ± 71	269 ± 45
	(650–1093)	(357–812)	(377–666)	(175–363)
Total	960 ± 92	760 ± 103	573 ± 65^∗†^	296 ± 39^∗†‡^
	(776–1144)	(583–997)	(442–703)	(217–376)

Two-way ANOVA: *P* < 0.001 between age groups, *P* = 0.034 between genders, and *P* = 0.234 for interaction between age and gender.				

Data expressed as mean ± SEM and 95% Confidence Intervals.

*Significantly different from children group.

^†^Significantly different from adolescent age group.

^‡^Significantly different from adult group.

^#^Significantly different from females of same age group.

^≠^Age × Gender = age and gender interaction.
